# Characterization of the mitochondrion genome of a *Chlorella vulgaris* strain isolated from rubber processing wastewater

**DOI:** 10.1080/23802359.2020.1789004

**Published:** 2020-07-10

**Authors:** Xiaowen Hu, Deguan Tan, Lili Fu, Xuepiao Sun, Jiaming Zhang

**Affiliations:** a Institute of Tropical Bioscience and Biotechnology, MOA Key Laboratory of Tropical Crops Biology and Genetic Resources, Hainan Bioenergy Center, Chinese Academy of Tropical Agricultural Sciences, Haikou, China; b Zhanjiang Experimental Station, CATAS, Zhanjiang, Guangdong Province, China; c Hainan Institute for Tropical Agricultural Resources, Chinese Academy of Tropical Agricultural Sciences, Haikou, China

**Keywords:** Chlorella, complete mitochondrial genome, wastewater, rubber

## Abstract

*Chlorella vulgaris* ITBBA3-12 was isolated from the rubber processing wastewater and has a role in wastewater purification. Its complete mitogenome contains 88754 bp, with a G + C content of 29.7%. A total of 64 genes were annotated, including 34 protein-coding genes, 27 tRNA genes, three rRNA (rrn23, rrn16, and rrn5). Phylogenetic analysis using the mitogenomes of Trebouxiophyceae species indicated that the strain ITBBA3-12 is closely related to *C. vulgaris* strain UTEX259 and NJ-7, and they clustered in the *Chlorella* lineage.

The genus *Chlorella* was first described by Beijerinck in 1890 (Beijerinck 1890), and has become one of the most investigated microalgae with diverse application prospects in the fields of bioenergy (El-Sheekh et al. 2019; Sakarika and Kornaros 2019), animal feed (Janczyk et al. 2007), human dietary (Draaisma et al. 2013; Torres-Tiji et al. 2020), and waste treatment (Pleissner et al. 2013; Gupta and Pawar 2018; Torres Franco et al. 2018). More than 100 species has been decribed since the establishment of the genus (Darienko et al. 2019). However, many species originally assigned to *Chlorella* based on morphological characters were re-classified into different lineages by phylogenetic analysis (Huss et al. 2002; Darienko et al. 2019). Genome-based phylogenetic analysis can produce well-resolved robust trees that reflect the overall relationship between highly related species (Alam et al. 2010), and is thus desired. *Chlorella vulgaris* Beijerinck is the type species of the genus *Chlorella*. Mitochondrion genomes of two strains NJ-7 and UTEX259 are available in GenBank database, and they have 10 kb difference in size and only 81% overall identifies, suggesting rapid evolution of the mitogenomes. In this study, we sequenced the complete mitochondrial genome of a microalgal strain morphologically similar to *C. vulgaris*. This strain was isolated from the rubber processing wastewater located in Danzhou city, Hainan Province, China with geospatial coordinates N19°30′59″, E 109°29′43″ and stored at the ClonBank of Institute of Tropical Bioscience and Biotechnology at −80 °C in 15% glycerol with accession number ITBBA3-12.

The genomic DNA was isolated as previously described (Ma et al. 2020; Yu et al. 2020), and sequenced using Illumina Hiseq 2500 and PacBio RSII platforms at Genoseq (Wuhan, China). The mitogenome was assembled using CANU (Koren et al. 2017) and GATK (Zhu et al. 2015), and deposited in the Genome Warehouse in National Genomics Data Center, Chinese Academy of Sciences, under accession number GWHANOW00000000. The circular mitochondrial genome has a length of 88754 bp, smaller as compared to the mitogenome of *C. vulgaris* strain UTEX259 (98062 bp, MK948103), but bigger than the mitogenome of *C. vulgaris* strain NJ-7 (87477 bp, NC_045362). The GC content is 29.7%, lower than the two sister strains (30.0%) and most other green algae. A total of 64 genes were annotated, including 34 protein-coding genes, 27 tRNA genes, three rRNA (rrn23, rrn16, and rrn5). The protein-coding genes include 13 for ribosomal proteins, nine for NAD(P)H-quinone oxidoreductases (nad), four for ATP synthases, three for coxs, one for cob, and three for intron-encoded endonucleases. Transfer RNA genes for all 20 amino acids were identified, in which tRNA-Met, tRNA-Leu are triplicated, and tRNA-Ser, tRNA-Arg, and tRNA-Gly are duplicated.

Phylogenetic analysis using the mitochondrial genomes of Trebouxiophyceae species indicated that strain ITBBA3-12 is closely related to *C. vulgaris* strain UTEX259 and NJ-7, and they clustered in the *Chlorella* lineage with 100% bootstrap support (Figure 1).

**Figure 1.**
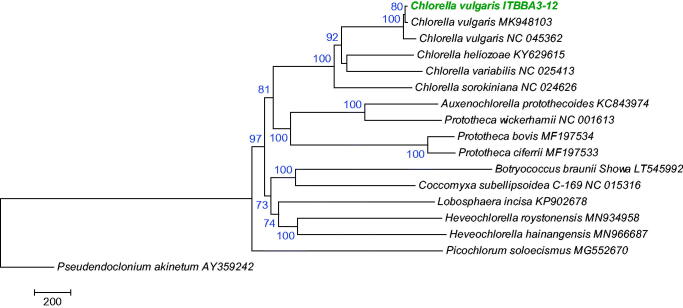
Evolutionary relationships of Trebouxiophyceae species based on mitogenomes. The tree was inferred using the Maximum Likelihood method and rooted with *Pseudendoclonium akinetum*. Bootstrap supports for clades (1000 replicates) are shown above the branches. Evolutionary analyses were conducted in MEGA7 (Kumar et al. 2016).

## Data Availability

The authors confirm that the data supporting the findings of this study are available within the article. The whole genome sequence data reported in this paper have been deposited in the Genome Warehouse in National Genomics Data Center (Members 2019), Beijing Institute of Genomics (China National Center for Bioinformation), Chinese Academy of Sciences, under accession number GWHANOW00000000 that is publicly accessible at https://bigd.big.ac.cn/gwh.
